# Individual Differences in Rhythmic Cortical Entrainment Correlate with Predictive Behavior in Sensorimotor Synchronization

**DOI:** 10.1038/srep20612

**Published:** 2016-02-05

**Authors:** Sylvie Nozaradan, Isabelle Peretz, Peter E. Keller

**Affiliations:** 1 Institute of Neuroscience (Ions), Université catholique de Louvain (UCL), Belgium; 2 International Laboratory for Brain, Music and Sound Research (BRAMS), Université de Montréal, Canada; 3 The MARCS Institute, Western Sydney University, Sydney, Australia; 4 Music Cognition & Action Group, Max Planck Institute for Human Cognitive & Brain Sciences, Leipzig, Germany

## Abstract

The current study aims at characterizing the mechanisms that allow humans to entrain the mind and body to incoming rhythmic sensory inputs in real time. We addressed this unresolved issue by examining the relationship between covert neural processes and overt behavior in the context of musical rhythm. We measured temporal prediction abilities, sensorimotor synchronization accuracy and neural entrainment to auditory rhythms as captured using an EEG frequency-tagging approach. Importantly, movement synchronization accuracy with a rhythmic beat could be explained by the amplitude of neural activity selectively locked with the beat period when listening to the rhythmic inputs. Furthermore, stronger endogenous neural entrainment at the beat frequency was associated with superior temporal prediction abilities. Together, these results reveal a direct link between cortical and behavioral measures of rhythmic entrainment, thus providing evidence that frequency-tagged brain activity has functional relevance for beat perception and synchronization.

The perception of temporal regularities in auditory rhythms is central to many human activities. In music and dance, for example, the synchronization of movements and sounds is facilitated by the perception of an underlying beat. A key aspect of musical beat is its *periodic* nature, yielding optimal predictability of upcoming events in a given sequence, thus allowing accurate and precise coordination of body movement with rhythmic events through time^1^,^2^.

A sense of beat can be induced by isochronous pulses (as with a metronome) and by complex rhythmic structures that do not systematically present a sound on each beat^3^, thus indicating that the beat is not itself a property of the stimulus but requires endogenous processes to emerge^4^,^5^,^6^. How the human brain builds an internal template of periodic beat from incoming rhythmic inputs remains an unresolved issue. Electroencephalographic (EEG) studies measuring steady-state evoked potentials (SSEPs; i.e., peaks at specific frequencies in the EEG spectrum; see^7^) have provided evidence for the entrainment of EEG cortical responses, i.e., frequency-tagged responses to the envelope of the rhythms (see^2^ for a review). These studies suggested that those SSEPs do not merely constitute a faithful encoding of the rhythm. Rather, the brain transformed the rhythmic input by amplifying some frequencies that coincided with the perceived beat frequencies, even in rhythms where sounds did not occur on each beat, i.e., syncopated rhythms^2^,^8^,^9^. Hence, SSEPs elicited at the beat frequency could in part reflect exogenous processes in response to the rhythmic input but also endogenous processes that play a role in predicting the timing of upcoming sounds, thereby facilitating the synchronization of movements with the beat.

The current study addressed the nature of this input-output transformation by investigating the relationship between individual differences in neural activity at beat frequency and behavioral measures of sensorimotor synchronization. To examine neural entrainment, SSEPs were measured in individuals with various levels of musical training as they listened to two auditory rhythmic patterns, one syncopated, thus relying on endogenous (top-down) beat generation, and the other unsyncopated, where beat generation could be driven exogenously by sounds marking each beat (Fig. 1). The rhythms were presented at different rates covering a range of musical tempi. Indeed, beat perception is optimal in a tempo range distributed around an inter-beat interval of 500 ms (2 Hz)^10^, between 400 ms and 900 ms (∼1 and 2.5 Hz)^11^.

Sensorimotor synchronization skills were subsequently examined in the same individuals using two paced finger-tapping tasks. One task assessed two different aspects of sensorimotor synchronization performance when tapping in time with the perceived beat of the syncopated and unsyncopated rhythm: accuracy (how close in time movements are to the beat onsets) and precision (how consistent these phase relations are across time). The other task assessed the ability to generate temporal predictions about upcoming beat locations while tapping in synchrony with tempo-changing auditory sequences. Interrelationships between measures obtained from these tasks were examined to test a general hypothesis about the role of neural entrainment in sensorimotor synchronization and a specific hypothesis concerning the role of endogenous entrainment in temporal prediction.

We hypothesize that neural entrainment is a basic prerequisite for sensorimotor synchronization^12^,^13^. Accordingly, if synchronization with the beat necessitates selective locking with the beat period, then the amplitude of SSEPs at the beat frequency during the listening task should be positively correlated with synchronization performance in the rhythmic beat tapping task across participants. Our second hypothesis is based on the assumption that neural entrainment to the beat reflects a mixture of exogenously (top-down) and endogenously (bottom-up) driven processes^14^, and that the endogenous component, in particular, supports temporal predictions that allow overt synchronization. We estimated the strength of neural entrainment to an endogenous beat by calculating the difference between the amplitude of beat-related SSEPs for the syncopated and unsyncopated rhythms. It was hypothesized that this measure of endogenous entrainment would be correlated with our behavioral measure of temporal prediction during sensorimotor synchronization. These behavior-brain relations were further characterized by testing the influence of tempo and the selectivity of SSEPs at beat as opposed to non-beat specific frequencies.

## Results

Twenty-two healthy volunteers took part in the study after providing written informed consent. Data from four of the 22 participants were not analyzed because of technical issues that occurred in different parts of the experiment. The remaining 18 participants presented a large range of individual differences in musical experience, according to criteria based on professional training plus the number of years of instrument playing/singing, summed over all instruments: 12.44 ± 6.79 [mean and standard deviation].

### Task 1. SSEPs

Evidence for the entrainment of EEG cortical responses, i.e., frequency-locked responses to the envelope of the rhythms, was evaluated by analyzing SSEP amplitudes elicited at beat frequency and at non-beat specific frequencies. SSEPs for the syncopated and unsyncoptated rhythms presented at the four tempi, averaged across participants (N = 18), are shown in Fig. 2. A one-sample t-test revealed that SSEP amplitudes at beat and non-beat specific frequencies (averaged across the non-beat SSEPs) were significantly greater than zero for the unsyncopated and syncopated rhythm at each tempo, thus indicating that significant SSEP signals emerged (Table 1).

Of primary relevance to our hypotheses, however, was the amplitude of SSEPs at the beat frequency (see Table 1). A 2 (beat vs. non beat frequencies) × 2 (rhythm) × 4 (tempo) ANOVA on the SSEPs (in μV) revealed a significant difference in amplitude at beat vs. non beat frequencies (F_1,17_ = 101.18, p < 0.0001, η^2^ = 0.85), corroborating previous evidence of a selectively enhanced brain response at the beat frequency when listening to these rhythms^2^,^9^. The ANOVA also revealed significant effect of ‘rhythm’, with greater amplitudes for the unsyncopated rhythm compared to the syncopated rhythm (F_1,17_ = 37.16, p < 0.0001, η^2^ = 0.68), which was qualified by a significant interaction between ‘frequency’ and ‘rhythm’ (F_1,17_ = 41.04, p < 0.0001, η^2^ = 0.70). This interaction indicated that a stronger response was elicited by the unsyncopated compared to the syncopated rhythm for the beat SSEP but not for the other SSEPs. The ANOVA also yielded a significant effect of tempo (F_2.01,34.16_ = 3.49, p = 0.04, η^2^ = 0.17), which was qualified by a significant interaction between ‘rhythm’ and ‘tempo’ (F_2.29,39.07_ = 8.18, p = 0.001, η^2^ = 0.32) and a significant interaction between the three factors (F_2.52,42.92_ = 10.23, p < 0.0001, η^2^ = 0.37). The beat SSEP elicited by the unsyncopated rhythm was greater at slow than at fast tempi (F_2.46,41.95_ = 7.06, p = 0.001, η^2^ = 0.29), whereas there was no significant difference across tempi for the syncopated rhythm (F_2.65,45.08_ = 2.08, p = 0.12, η^2^ = 0.11). These differential effects of tempo on neural entrainment to the beat in syncopated and unsyncopated rhythms suggest a qualitative difference in processing for the two types of rhythm.

### Task 2. Performance of movement synchronization with the beat

Data for synchronization accuracy and precision in the tapping task for the two rhythms at the four tempi are reported in Table 2, and data for individual participants, averaged across conditions, can be seen in Fig. 3. Data from two out of the eighteen participants of task 2 were excluded of task 2 because these individuals tapped at the theoretical beat unit only in two out of the eight conditions, thus apparently using something other than the instructed strategy more often than not.

Synchronization accuracy was assessed based on how close each participant’s finger tap times were to corresponding beat onsets, with accuracy being high to the extent that mean signed asynchrony between taps and beats is close to 0 ms. As is typically observed in sensorimotor synchronization studies^15^,^16^, most participants produced a negative mean asynchrony, indicating that, on average, their taps preceded beat onsets, especially at slow tempi (Table 2 for N = 16). There were, however, large individual differences in mean asynchrony, with some participants tapping up to 180 ms before beat onsets, on average, while others producing mean asynchronies close to 0 ms (Fig. 3A). A 2 (rhythm) × 4 (tempo) ANOVA on mean assigned asynchronies yielded a significant main effect of rhythm (F_1,15_ = 10.80, p = 0.005, η^2^ = 0.41), a significant main effect of tempo (F_2.12,31.91_ = 11.73, p < 0.0001, η^2^ = 0.43), and a significant interaction between ‘rhythm’ and ‘tempo’ (F_2.16,32.48_ = 4.92, p = 0.01, η^2^ = 0.24). Specifically, the mean signed asynchronies were found to be relatively close to zero and not affected much by tempo for the syncopated rhythm but was large and negative for the unsyncopated rhythm at slow tempi and then close to zero at fast tempi. The difference in asynchrony for the two rhythms may be due to less anticipation of beat onsets for the syncopated rhythm at slow tempi^16^ leading to a greater proportion of relatively late taps (only 7/16 participants with negative mean asynchrony for the syncopated rhythm at slow tempi, compared to 15/16 participants for the unsyncopated rhythm).

The precision of synchronization was assessed based on the mean circular variance of taps relative to beat onsets. Circular variance can range from 0 to 1^17^, with variance being low, and hence precision high, to the extent that taps are concentrated around beat onsets. As for accuracy, these values were also consistent with the values typically obtained in tapping studies^16^,^17^,^18^ (Table 2). An ANOVA revealed no significant difference between the two rhythms (F_1,15_ = 1.04, p = 0.32, η^2^ = 0.06) but a significant difference across the four tempi (F_2.12,31.81_ = 21.97, p < 0.0001, η^2^ = 0.59). These results indicate that precision was greater at slow than at fast tempi. The interaction between the factors ‘rhythm’ and ‘tempo’ was not significant (F_2.28,34.23_ = 2.46, p = 0.09, η^2^ = 0.14). Note that the finding that the two rhythms do not differ in precision (despite the difference in accuracy) corroborates the above account of the difference between asynchrony for the two rhythms at the slow tempi. Although asynchronies were on average closer to zero for the syncopated rhythm at slow tempi, successive asynchronies varied between negative (early) and positive (late) values.

### Task 3. Temporal prediction

Temporal prediction indices were obtained for 15 out of the original 18 participants in a task requiring synchronization with tempo-changing pacing sequences (see Materials and Methods). To estimate the degree to which individuals were predicting vs. tracking tempo changes in the pacing signals, a prediction-tracking index was computed based on lag0 and lag-1 cross-correlations between inter-tap intervals (ITI), and the pacing signal IOIs, in each trial. Indices greater than 1 reflect a stronger tendency to predict than to track tempo changes. Temporal prediction indices (Fig. 3B), which ranged from 1.007 to 1.110 (1.06 ± 0.02 [mean and standard deviation]), as well as synchronization accuracy (mean asynchrony = −36.04 ± 15.40 ms) and precision (standard deviation of asynchronies = 41.66 ± 8.38 ms), were commensurate with results observed in previous studies using this task^18^,^19^,^20^. Consistent with previous work^20^, temporal prediction indices increased with years of musical training (R = 0.571, p = 0.026). The finding that temporal prediction indices were nevertheless greater than 1 indicates that all participants, despite individual differences, engaged in more prediction than tracking when tapping in time with the tempo changes.

### Relations between SSEPs and behavioral measures

Separate multiple regression analyses were conducted to test our two hypotheses concerning the relationship between individual differences in SSEPs reflecting neural entrainment to the beat and behavioral measures of sensorimotor synchronization skill. Synchronization accuracy and precision in the beat tapping task were the dependent variables in the first and second regression analyses (N = 16) and our behavioral index of temporal prediction during synchronization with tempo-changing sequences was the dependent variable in the third regression analysis (N = 15). Predictor variables in each analysis included (i) the average SSEP amplitude at the beat frequency, (ii) our measure of the strength of neural entrainment to an endogenous beat (the difference between the amplitude of SSEPs at beat frequency for the syncopated and unsyncopated rhythms), and (iii) years of musical training.

When significant, these regression analyses were followed up with further regression analyses performed separately for the slow (mean of 0.6 and 1.25 Hz) and fast (mean of 2.5 and 3.8 Hz) tempi, in order to assess how these behavior-brain relations were influenced by the tempo of the rhythmic sequence. Moreover, to test the selectivity of these behavior-brain relations for SSEPs elicited at beat frequency specifically, follow-up analyses were also performed separately for the beat as opposed to the non-beat specific SSEPs.

#### Relations with synchronization accuracy

The first analysis yielded a model with R^2^ = 0.64 (F[3,12] = 7.28, p = 0.005), with average SSEP amplitude at the beat frequency (Beta = 0.49, p = 0.02) emerging as the only significant unique predictor of mean asynchrony. A scatterplot showing the positive correlation between SSEP amplitude at beat frequency and mean asynchrony is shown in Fig. 3A. In support of our general hypothesis about the role of neural entrainment, stronger entrainment of neural activity to the beat was thus related to greater accuracy (mean asynchrony closer to zero) in synchronizing movements with the beat.

#### Relations with synchronization precision

The second analysis yielded a model with R^2^ = 0.45 (F[3,12] = 3.35, p = 0.06). In contrast with accuracy, the precision of tapping on the beat does not correlate with our measures of neural entrainment to the beat (Beta = 0.12, p = 0.58 for the predictor ‘average SSEP amplitude at beat frequency’ and Beta = −0.20, p = 0.39 for the predictor ‘neural entrainment to an endogenous beat’). Precision did, however, correlate with years of musical training, with higher training being associated with lower circular variance (Beta = −0.516, p = 0.049).

#### Relations with temporal prediction

The third analysis yielded a model with R^2^ = 0.67 ([F3,11] = 7.57, p = 0.005), with our measure of the strength of neural entrainment to an endogenous beat emerging as the only significant unique predictor of temporal prediction indices (Beta = 0.64, p = 0.007). A scatterplot showing the positive correlation between the strength of endogenous beat-related neural entrainment and prediction indices is shown in Fig. 3B. In support of our specific hypothesis about endogenous neural entrainment, individuals who were relatively good at predicting upcoming events in temporally changing sequences also displayed a greater amount of entrainment of neural populations under conditions where the beat was not marked by physical sound onsets during passive listening (i.e. these participants relied less on external information to perceive the beat).

#### Influence of tempo

The measures of tapping asynchrony (task 2) showed a tendency towards positive correlation with the beat SSEP amplitude elicited at slow tempi, but this correlation was not significant (R = 0.46, p = 0.07). This tendency was not present at fast tempi (R = 0.05, p = 0.85). More interestingly, a multiple linear regression analysis with the prediction index (task 3) as the dependent variable and the measure of neural entrainment to an endogenous beat at slow and fast tempi as predictors yielded a model with R^2^ = 0.61 (F2,12 = 9.51, p = 0.003), with the measure of neural entrainment at slow tempo (Beta = 0.76, p = 0.001) emerging as the only significant unique predictor of temporal prediction indices. Together, these results suggest that the behavior-brain relation found for beat processing and sensorimotor synchronization does not hold for beat rates higher than 2 Hz using these rhythms.

#### Selectivity for the beat SSEP

When correlating the non-beat specific SSEP values (standardized values) with the mean asynchrony (across all tempi) in tapping the beat (task 2), the non-beat specific SSEPs were found to be negatively correlated with the mean asynchrony in tapping (R = −0.65, p = 0.005). This indicates that SSEPs at non-beat frequencies were overall inversely related to beat tapping behavior. When testing this correlation separately for slow and fast tempi, there was no significant correlation found at slow or fast tempi (R = −0.46, p = 0.07 and R = −0.05, p = 0.85 respectively).

A multiple linear regression analysis with the prediction index (task 3) as dependent variable and the amplitudes averaged across the non-beat specific SSEPs for slow and fast tempi as predictors yielded a model with R^2^ = 0.31 (F[2,12] = 2.73, p = 0.10), indicating that, as opposed to the beat SSEP, the temporal prediction index does not correlate with the non-beat specific activity. However, partial correlations calculated from this model nevertheless indicated a negative correlation between the prediction index and non-beat specific SSEPs at fast tempi (Beta = −0.54, p = 0.04) but no correlation at slow tempi (Beta = −0.04, p = 0.85).

## Discussion

To what extent are overt entrainment behaviors related to covert neural entrainment? To address this question, we conducted a combined behavioral-EEG study using three separate tasks that allowed us to compare individual indices of temporal prediction, the accuracy and precision of sensorimotor synchronization, and neural activity in response to a syncopated and an unsyncopated musical rhythm. Based on the frequency-tagged responses (SSEPs) to these rhythms, we computed a general index of neural entrainment to the beat and a specific index of neural activity to an endogenous beat for each individual participant. For the latter measure, top-down components of beat perception were isolated from bottom-up processes driven by acoustic input by contrasting neural activity measured at the beat frequency in response to the syncopated vs. unsyncopated rhythm.

The results provide objective evidence for two ways in which covert neural entrainment facilitates overt sensorimotor synchronization with auditory rhythms. First, we show that our general index of neural entrainment—the amplitude of neural activity selectively locked to the beat period—could explain individual differences in movement synchronization accuracy with the beat. This finding establishes a link between the overall strength of neural period-locking and basic sensorimotor synchronization skills. Second, we found that stronger endogenous neural entrainment to the beat was associated with superior temporal prediction abilities. This indicates that the endogenous component of covert neural processes, in particular, supports temporal predictions that allow individuals to anticipate upcoming events within a sequence, and thereby to engage in preparatory motor planning that enables the overt synchronization of movements with complex temporal structures.

The exact nature of the neurophysiological substrates of SSEPs elicited at beat frequency when listening to auditory rhythms remains unclear. Indeed, whether these activities result from ongoing neural activities resonating at the frequency of the stimulation^21^,^22^,^23^ or whether they result from the linear superposition of independent transient event-related potentials elicited by the rhythmic stimulus^7^,^24^ is a matter of debate. Irrespective of the outcome of this debate, the responses captured with surface EEG at the beat frequency in the current study are likely to sample only a portion of the brain activity involved when perceiving a beat from rhythms. SSEPs at beat frequency are assumed to contain not only beat-related neural activity but also overlapping activity at harmonics related to lower frequencies. Nevertheless, by showing a positive correlation between such overall activity and behavioral measures of sensorimotor synchronization and temporal prediction, the current results provide evidence for the functional relevance of this brain activity related to rhythm processing. In addition, this behavior-brain relation is not general to the overall neural activities elicited by the rhythms but is specific to the beat frequency. Importantly, the observed tendency toward a negative correlation between behavioral measures and non-beat SSEPs indicates that the behavior-brain relation is *selective* to the beat frequency to the detriment of the activities elicited by the rhythms at the other frequencies. Given the increasing usage of SSEP measures in research on rhythm processing^25^,^26^,^27^,^28^, these observations advance our understanding of the nature of these neural activities.

Our findings have theoretical implications for understanding the functional significance of neural entrainment and the functional basis of individual differences in rhythm perception and sensorimotor synchronization skills. With regard to the functional role of neural entrainment, the finding that neural processes are linked with overt behaviors through temporal prediction is consistent with predictive coding models of brain function^29^,^30^,^31^,^32^,^33^. According to these models, neural populations at relatively high cortical levels continuously generate internal templates based on past experience in order to facilitate the efficiency of information processing by providing (top-down) predictions about incoming sensory input to early sensory areas. A significant body of research has found evidence for predictive coding in perception, and there is increasing interest in understanding the role of predictive coding in action control^34^,^35^. During beat entrainment in the context of sensorimotor synchronization tasks, for instance, the corresponding tapping behavior has been shown to produce cyclic fluctuations in sensory gain that are locked to individual movements, suggesting that top-down signals linked to rhythmic motor execution may tune representations of incoming sensory information^36^,^37^,^38^. In the current study, the positive relationship between the endogenous neural component and the index of temporal prediction that we report here contributes to this research effort by providing support for the predictive coding model in the context of sensorimotor synchronization with musical rhythms^29^.

The present findings underscore the importance of combining neural data with appropriate behavioral measures in order to gain insight on the functions supported by beat-related neural entrainment. We captured cortical responses to musical rhythms using frequency tagging^2^ because this approach permits neural responses to be measured without the need for explicit behavioral responses or overt movement, which could potentially bias these perceptual measures^39^. While the frequency-tagging approach has proven to be revealing about the conditions that give rise to beat-related neural activities, the functional relevance of these oscillations had not previously been addressed directly. This issue was critical to resolve because any purely neural data must be interpreted with caution in the absence of behavioral evidence, as neural and behavioral measures can lead to divergent conclusions under some conditions^40^,^41^. Here, we provide an answer to the question of functional significance by showing that beat-related SSEPs are linked to individual differences in human behavior.

An additional advantage of the approach taken in the current study is that it allowed us to gain insight into the neural bases of individual variability in beat perception and sensorimotor synchronization. These behaviors are remarkable in that they appear to be spontaneous in humans and ubiquitous across cultures^42^. However, there are numerous anecdotal reports and a few recent studies showcasing the large individual differences in the ability to perceive a beat, ranging from acute processing in musicians to severe impairments associated with beat deafness^43^,^44^,^45^,^46^. Previous work has taken various approaches to clarify the neural mechanisms explaining such individual differences. For instance, correlations have been found between the ability to synchronize to a beat and the functional involvement of auditory and motor regions of the brain during beat perception^43^,^47^. In another study, measures of finger-tapping performance obtained in synchronization-continuation tapping tasks were shown to relate positively to gray and white matter volume in frontal cortex^48^. Furthermore, the consistency of brainstem responses evoked by sounds has been related to the variability in inter-tap intervals when synchronizing with regular sound sequences^49^. In line with these studies, the current work takes an important step forward by demonstrating that the *endogenous* processes of beat perception and temporal prediction are linked to the ability of an individual’s neural system to dynamically entrain to the beat. Our findings thus identify a high-level cortical process that may call upon the structures and mechanisms identified in previous work.

Whether neural populations dedicated specifically to auditory processing or interconnected auditory-motor networks contributed to this cortical process remains to be investigated. For example, given that auditory and motor/premotor cortex are functionally connected during synchronized tapping tasks (see e.g.^50^,^51^), characteristics of neural activity in these different regions may be tied to synchronization ability. Moreover, a number of non-human primate studies^52^,^53^,^54^ have shown that premotor areas and the basal ganglia in monkeys performing a synchronization-continuation task exhibit interval tuning to the tapping tempo. This tuning phenomenon at the single cell level could be related to the global beat-related SSEPs observed in the current study. However, the low spatial resolution inherent to surface EEG prevents us from characterizing precisely the distributed neural network involved in the behavior-brain relation observed here. Future studies using the same approach but sampling brain activity through other techniques, such as the invasive recording of local field potentials in human patients, could address this crucial question more adequately^55^.

Interestingly, our measure of musical training was positively correlated with the index of temporal prediction^20^, but not with the neural measures. While intriguing, this null result highlights the complexity and the wide range of abilities related to rhythm processing. Future studies addressing this question more comprehensively are needed to clarify how musicians’ brains develop through daily training. For example, a more specific characterization of the individual’s musical training based on the learnt musical instrument (e.g., instruments that typically play a rhythmic vs. melodic role) or the practiced musical style (e.g., groove-based music with a salient beat vs. more freely timed music) might help to reveal finer processes that shape underlying neural activity by long-term musical expertise^56^.

Another intriguing result is the dissociation between the two aspects – accuracy and precision– of behavioral synchronization as measured in task 2 in their relationship with the measures of neural entrainment (task 1). Specifically, the amplitude of SSEPs was related to accuracy (how close in time movements are to synchronization targets) rather than precision (how consistent these phase relations are across time) when tapping the beat of the rhythms, suggesting that synchronization accuracy and precision are supported by neural mechanisms that are distinctly involved when processing the beat of an auditory rhythm. However, it should be noted that these tapping measures were only based on one 40-s tapping trial per sequence. While this limited sampling yielded a positive brain-behavior relation, more extensive sampling, as in task 3, will allow this relation to be characterized more specifically in future work.

The observed behavior-brain relation was also characterized by a bias towards slower beat tempo (under 2 Hz), suggesting that temporal prediction and synchronization accuracy operate differently at higher tempi. This result could be related to the tempo preference region with greatest beat saliency usually observed between 1 and 2.5 Hz in music^10^,^11^. Moreover, emphasis on higher-order prediction could be stronger at faster tempi, where beats are grouped according to higher-order metric units corresponding to relatively long time spans in the sequence (e.g. measures or bars) and predictions are made at that level (in addition to lower levels in the metric hierarchy).

Going beyond the specific context of musical beat perception and synchronization, temporal coordination with environmental events is a capacity that is not restricted to humans. Rhythmic coordination is concomitant to multiple biologically vital behaviors (e.g. rhythmic hooting and coordinated group displays in apes^57^). This raises questions about the generality of the relationship between covert neural entrainment and overt movement synchronization, which is an issue that has broader implications concerning the evolutionary origins of human musical behavior. For this reason, there has been growing interest in the sensorimotor synchronization abilities of non-human animals^26^,^40^,^58^. This work has revealed that non-human animals do not exhibit behavioral entrainment to the beat with the same degree of accuracy and flexibility observed in humans^26^,^40^,^58^. However, it cannot be excluded that similar profiles of neural responses to auditory rhythms will be found in these non-human species and in humans^26^. In fact, such similarity between human and non-human species may reveal a common basis of low level processing of exogenous rhythmic sequences. Importantly, although similar profiles of neural activity may be displayed across distinct species, the link with behavior may not be shared across species. Rather, it may be the case that humans are unique in exploiting this form of auditory processing to drive sensorimotor behavior requiring fine-grained temporal prediction. The current findings are hence key to the extent that they provide evidence for a positive link with behavior, thus pointing to a possibly fundamental difference between humans and other species.

## Materials And Methods

### Participants

Twenty-two healthy individuals took part in the study (15 females, all right-handed, mean age 24 ± 3 years). None had prior experience with the tapping task, or the rhythms used in the present study. They had no history of hearing impairment or neurological or psychiatric disorders, and were not taking any drug at the time of the experiment. The study was approved by the ethics committee of the University of Leipzig (Germany) and carried out in accordance with the approved guidelines.

### Task 1. EEG recording while listening to syncopated and unsyncopated rhythms

#### Auditory stimuli

The auditory stimuli were created in Audacity 1.2.6 ( http://audacity.sourceforge.net/) and presented binaurally through earphones at a comfortable listening level using Presentation software (NBS, Berkeley, USA).

**Figure 1:**
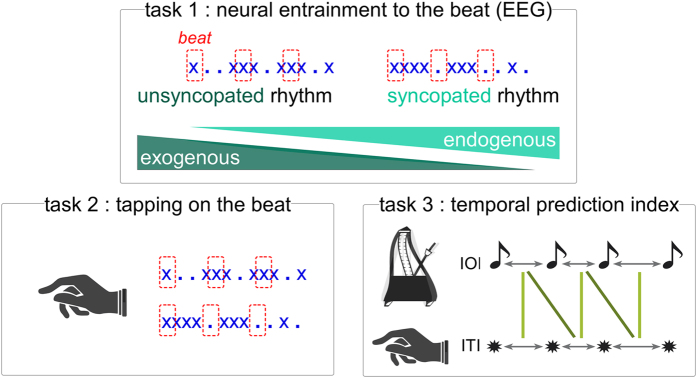
Experimental design. SSEPs were measured in response to two auditory rhythms, one syncopated (i.e., with some beats coinciding with silences, thus requiring endogenous processes to perceive a beat) and the other unsyncopated (i.e., with tones coinciding with all beats, thus allowing exogenously driven beat perception) (task 1). Participants were afterwards asked to tap the beat of the rhythms (task 2). In a later session, the same individuals completed a finger-tapping task assessing the degree to which they predicted timing variations while synchronizing with tempo-changing auditory sequences (task 3). This measure was obtained by comparing the inter-onset intervals (IOI) between tones in the sequence with the inter-tap intervals (ITI) at lag 0 (vertical lines) and lag -1 (oblique lines).

The stimuli consisted of two different rhythms looped continuously for 33 s (Fig. 1). The structure of the rhythms was based on the alternation of 12 events, consisting of sounds and silent intervals (the sounds consisted of 1000 Hz pure tones, with 10 ms rise and fall times and had variable duration depending on tempo as described below). Motivated by the work of Povel and Essens^59^, the rhythms were designed to induce the perception of a beat based on a preference for grouping by 4 events, at points represented in Fig. 1 (i.e., either silent or tone events)^9^ with 3 such beats per cycle. Accordingly, the first rhythm can thus be considered as unsyncopated, as a tone coincides with every beat, whereas the second rhythm is considered as syncopated, as it includes instances where a tone occurs between beats and is followed by silence on the next beat.

To test whether the hypothesized neural entrainment to the beat was robust across distinct musical tempi, participants were presented with the 2 rhythms at 4 different tempi, from slow to fast. At the slowest tempo, the individual events of the rhythms lasted 400 ms (2.5 Hz); at tempo 2, 200 ms (5 Hz); at tempo 3, 100 ms (10 Hz); and at the fastest tempo, 66 ms (15 Hz). Thus, the duration of one cycle of the rhythms at each of these tempi corresponded to 9600 ms for tempo 1, 2400 ms for tempo 2, 1200 ms for tempo 3 and 792 ms for tempo 4. Since the perceived beat is expected to coincide with the grouping by four events in the rhythms^9^,^59^, the duration of one beat cycle at each of these tempi corresponded to 1600 ms (0.6 Hz) for tempo 1, 800 ms (1.25 Hz) for tempo 2, 400 ms (2.5 Hz) for tempo 3 and 264 ms (3.8 Hz) for tempo 4.

#### Sound analysis

**Figure 2:**
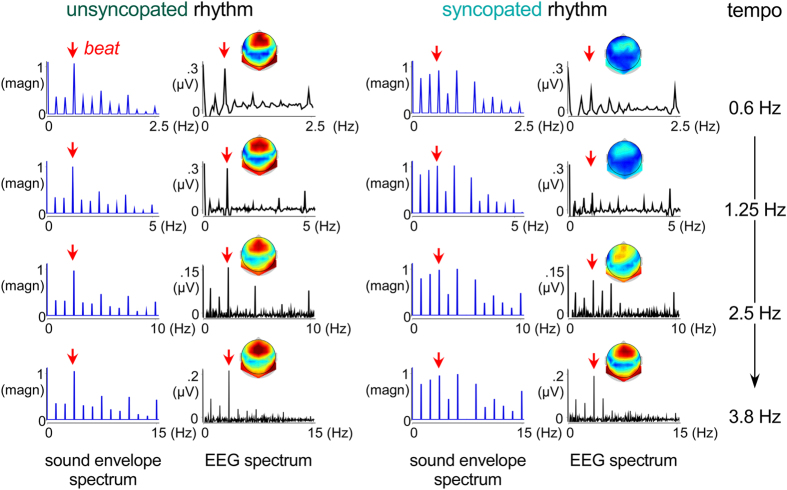
Spectrum of the stimulus rhythms’ envelope (in blue) and corresponding EEG (in black, averaged across participants and all channels) as recorded from task 1. The unsyncopated and syncopated rhythms were played at four distinct tempi, from slow (top) to fast (bottom), with beat frequencies at 0.6 Hz, 1.25 Hz, 2.5 Hz and 3.8 Hz, respectively (arrows). Note that at all tempi, the spectrum corresponding to the unsyncopated rhythm shows a prominent peak in acoustic energy at the beat frequency, in contrast to the syncopated rhythm. The topographical distribution of this activity was generally frontocentral for all rhythms and tempi (color scale ranges correspond to the y axis, with hot colors indicating high values).

**Figure 3:**
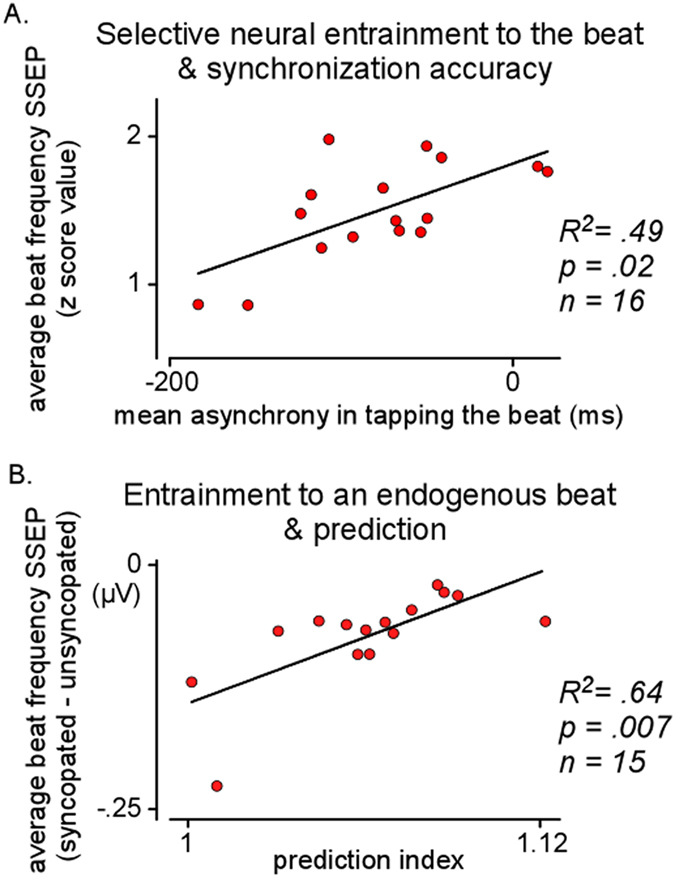
Scatterplots showing individual indices as obtained from task 1 (selective neural entrainment at the beat frequency and neural entrainment to an endogenous beat), task 2 (synchronization accuracy) and task 3 (temporal prediction). Panel A illustrates that synchronization accuracy improves (mean signed asynchronies closer to zero when synchronizing to the beat) with increasing strength of selective neural entrainment to the beat. Panel B illustrates that individuals with high prediction indices show high neural entrainment to an endogenous beat. Note that 3 from the 18 participants did not return for task 3 and that 2 from the 18 participants were excluded from task 2 analyses because they did not tap as instructed.

To determine the frequencies at which steady-state evoked potentials (SSEPs) were expected to be elicited in the recorded EEG signals, the temporal envelope of the 33-s sound rhythms was extracted using a Hilbert function, yielding a time-varying estimate of the instantaneous amplitude of the sound envelope, as implemented in Matlab. The obtained waveforms were then transformed in the frequency domain using a discrete Fourier transform, yielding a frequency spectrum of acoustic energy. As shown in Fig. 2, the envelopes of the rhythm consisted of 12 distinct frequencies ranging from the frequency corresponding to the period of the entire rhythm to the frequency corresponding to the period of the unitary event at each tempo of presentation.

#### Experimental conditions

The two rhythms were presented at the four tempi (i.e. eight stimuli) in eight separate blocks run across two sessions on separate days. The order of the blocks was identical for each participant (unsyncopated rhythm, tempo 0.6 Hz, 1.25 Hz, 2.5 Hz and 3.8 Hz, and then syncopated rhythm, from tempo 0.6 Hz to tempo 3.8 Hz).

In each block, the 33-s auditory stimulus was repeated 11 times. Each rhythm was presented 3-s after the participant pressed a key to start the trial. During the first 10 trials of each block, participants were asked to listen carefully to the stimulus in order to detect the occurrence of a brief acceleration (the interval between two successive events reduced by 20%) or deceleration (the interval between two successive events increased by 20%) of tempo, inserted at a random position in two of the trials within the block. The participants were instructed to report whether they detected a change in tempo at the end of each trial. This task ensured that participants focused their attention on the temporal aspects of the auditory stimuli. The trials containing tempo changes were excluded from further analyses.

During the 11^th^ trial of each block, participants were asked to perform a tapping task that assessed their ability to move in synchrony with the perceived beat for each rhythm (see description of task 2 below).

#### EEG recording

Participants were comfortably seated in a chair with the head resting on a support. They were instructed to relax, avoid any unnecessary head or body movement and keep their eyes fixated on a point displayed on a monitor in front of them. The electroencephalogram (EEG) was recorded using 64 Ag-AgCl electrodes placed on the scalp according to the International 10/10 system. Vertical and horizontal eye movements were monitored using four additional electrodes placed on the outer canthus of each eye and on the inferior and superior areas of the left orbit. Electrode impedances were kept below 10 kΩ. The signals were amplified, low-pass filtered at 512 Hz, digitized using a sampling rate of 1024 Hz and referenced to an average reference (64-channel high-speed amplifier, Biosemi, The Netherlands).

#### EEG analysis

The continuous EEG recordings were filtered using a 0.1-Hz high-pass Butterworth zero-phase filter to remove very slow drifts in the recorded signals. Epochs lasting 32 s were obtained by segmenting the recordings from + 1 to + 33 s relative to the onset of the auditory stimulus. The EEG recorded during the first second of each epoch was removed (i) to discard the transient auditory evoked potentials related to stimulus onset^8^,^9^,^60^, (ii) because SSEPs require several cycles of stimulation to be entrained^7^ and (iii) because several beat cycles are required to elicit stable beat perception^16^.

EEG epochs were averaged across trials for each participant and condition. The time-domain averaging procedure was used to enhance the signal-to-noise ratio of EEG activity time-locked to the rhythms. The obtained average waveforms were then transformed in the frequency domain using a discrete Fourier transform, yielding a frequency spectrum of signal amplitude (μV) ranging from 0 to 512 Hz with a frequency resolution of 0.031 Hz per bin in the spectrum. These EEG processing steps were carried out using Letswave5 ( http://nocions.webnode.com/) and Matlab (The MathWorks, USA).

Within the obtained frequency spectra, signal amplitude may be expected to correspond to the sum of (i) stimulus-induced SSEPs and (ii) unrelated residual background noise (e.g., due to spontaneous EEG activity, muscle activity or eye movements). To obtain valid estimates of the SSEPs, the contribution of this noise was removed by subtracting, at each point of the frequency spectra, the average amplitude measured at neighboring frequency bins (2 frequency bins ranging from −0.15 to −0.09 Hz and from + 0.09 to + 0.15 Hz relative to each frequency bin) (see^61^,^62^). To allow SSEP amplitude to be compared across participants while avoiding electrode selection bias, SSEP magnitudes were averaged across all scalp electrodes for each rhythm and tempo for each participant (Fig. 2)^8^,^9^. The global mean amplitude measure is based on the assumption that the neural activity associated to beat processing originate from a large network of distant brain regions (including for instance temporal but also frontal or parietal areas; see e.g.^12^) and might thus spread over topographical regions. Hence, the 64 channels were collapsed to characterize global EEG activity at the beat frequency and compare SSEP amplitudes across participants while avoiding electrode selection bias.

To test our hypotheses about relations between neural entrainment to the beat and behavioral measures of sensorimotor synchronization and temporal prediction, two indices of neural entrainment to the beat were derived from EEG data of each individual. First, a general index of *selective neural entrainment at beat frequency* was obtained for each rhythm and tempo by expressing the amplitude of the SSEP elicited at the expected beat frequency (x) as a z-score value: z = (x–μ)/σ, where μ and σ correspond to the mean and standard deviation of the magnitudes obtained across the different peaks^9^,^25^. This procedure allowed the assessment of how the SSEP elicited at beat frequency stood out relative to the entire set of SSEPs for each rhythm and tempo in a given individual^9^. Averaging across rhythms and tempi provides an index of mean individual selective neural entrainment to the beat.

Second, to address our specific hypothesis concerning the role of the endogenous component of the beat-related SSEPs in temporal prediction, an index of *neural entrainment to an endogenous beat* was obtained by subtracting, for each tempo and participant, the amplitude obtained at beat frequency in the unsyncopated rhythm from the amplitude obtained in the syncopated rhythm. The logic behind this subtraction is based on the assumption that beat-related SSEPs for syncopated and unsyncopated rhythms reflect exogenous and endogenous processes to differing degrees: SSEPs for syncopated rhythms largely reflect endogenous processes, while beat-related SSEPs for unsyncopated largely reflect exogenous processes (because 1/3 vs. 3/3 beats coincide with a sound in the syncopated rhythm and unsyncopated rhythm, respectively) (Fig. 1). Hence, subtracting beat-related SSEPs for the unsyncopated rhythm from those for the syncopated rhythm allows the relative strength of endogenous processes involved in inducing an internal beat template to be estimated (i.e. the higher the resulting score, the more endogenous entrainment).

### Task 2. Sensorimotor synchronization with the beat in syncopated and unsyncopated rhythms

#### Beat tapping procedure

During the 11^th^ trial of each block, participants were asked to tap the index finger of their right hand in time with the perceived periodic beat of the stimulus rhythms. Participants received the instruction for this task before the recording session. Specifically, they were instructed to tap periodically on the rhythms at the rate that feels natural. They were instructed to start tapping as soon as possible after the start of the trial, and to continue tapping until the rhythm ended. An example of periodic tapping with the rhythm was given by the experimenter tapping for a few cycles of each of the two rhythms on the theoretical beat location (corresponding to the red squares in Fig. 1). Tapping was performed on a custom-built response pad with small up and down movements of the hand while the forearm and elbow were fixed on an armrest cushion. Taps did not trigger feedback sounds. The latency of each finger impact on the pad’s surface was registered with millisecond accuracy and recorded in the Presentation program that controlled stimulus delivery.

#### Beat tapping analysis

The accuracy and precision of sensorimotor synchronization was computed based on the tap timing data for each rhythmic stimulus using circular statistics (e.g.^18^,^63^), as implemented in a Matlab toolbox^17^. By aligning the time series of tapping period to a window corresponding to the beat period at each tempo, all finger taps within each trial were mapped onto a circular scale, i.e., a unit circle ranging from 0 to 2 π in radians (or 0–360 degrees) with the tapping target (the beat onset) located at 0. Importantly, as not all participants tapped at the theoretical beat levels in all conditions (they sometimes either tapped at the beat subdivision or at the bar level), the mapping was done using the individual participants’ target beat intervals (which were inferred based on their inter-tap intervals) rather than theoretical beat units. The degree to which participants tapped at target tempo was measured by calculating the root mean square error (RMSE) of tapped tempo versus theoretical beat unit. Participants with high RSME values, indicating that they did not tap at the theoretical beat unit in the majority of the conditions were excluded from further analysis.

Accuracy at tapping was assessed by calculating the mean resultant direction of the vector representing each remaining participant’s taps in each rhythm × tempo condition. These values were then converted from radians to asynchronies (in ms), and the signed value was taken to indicate how close the participant’s finger taps were to the corresponding beats on average. Hence, scores closer to zero indicate higher accuracy.

The precision of sensorimotor synchronization was assessed by calculating the mean resultant length of each vector, which is inversely related to the circular variance. This way, the variance across taps is only interpreted proportionally to its target period, regardless of the duration of the beat period. Circular variance ranges between 0 and 1 17, with higher scores indicating higher variance (greater spread in tap times relative to beat onsets) and hence lower precision.

### Task 3. Temporal prediction during sensorimotor synchronization

This task, taken from^18^, was performed on a separate day several weeks after tasks 1 and 2. Only 15 out of the 18 participants returned for this task.

#### Materials and Procedure

The task involved tapping in time with auditory sequences that contained tempo changes resembling those found in music (i.e., accelerando and ritardando), as used in^18^. The procedure consisted of 12 40-s trials in which the sequences progressed through both accelerations and decelerations following a sinusoidal function of inter-onset intervals, which increased or decreased with step sizes varying between 11 ms and 64 ms, within an outside range of 400 ms (2.5 Hz) and 600 ms (1.6 Hz). There were six arrangements, with each one differing in terms of the time points of switches between acceleration and deceleration throughout the sequence. Each event in the auditory signal consisted of a sampled bell sound presented over headphones (Sennheiser HD 280 Professional). The participant was instructed to tap their right index finger on a MIDI percussion pad (Roland Handsonic HPD-10) in synchrony with each sequence and to “keep in time with the changing tempo”. Taps did not trigger feedback sounds. Stimulus presentation and response registration were controlled by a computer program written in MAX/MSP.

#### Estimating temporal prediction

To estimate the degree to which individuals were predicting *vs.* tracking tempo changes in the pacing signals, a prediction-tracking index were computed based on lag0 and lag-1 cross-correlations between inter-tap intervals (ITI) and the pacing signal IOIs in each trial (Fig. 1). The lag-0 cross-correlation between the IOIs and ITIs is high to the extent that an individual anticipates the tempo changes, while the lag-1 cross-correlation is high to the extent that he or she tracks the tempo changes. The ratio of lag0 over lag-1 thus reflects whether an individual is mainly predicting (ratio >1) or tracking (ratio <1) ongoing tempo changes (see^49^).

### Statistical analyses of the behavior-brain relations

The relations between SSEPs and behavioral measures were estimated using multiple linear regression analyses, which corrected for the number of predictors (as implemented in SPSS Statistics 21.0, IBM, Armonk, NY, USA).

## Additional Information

**How to cite this article**: Nozaradan, S. *et al.* Individual Differences in Rhythmic Cortical Entrainment Correlate with Predictive Behavior in Sensorimotor Synchronization. *Sci. Rep.* **6**, 20612; doi: 10.1038/srep20612 (2016).

## Figures and Tables

**Table 1:** SSEPs at beat and non-beat frequencies for unsyncopated and syncopated rhythms at the four tempi.

SSEP at beat frequency	0.6 Hz tempo	1.25 Hz tempo	2.5 Hz tempo	3.8 Hz tempo
unsyncopated rhythm	0.21 ± 0.11 μV t_17_ = 7.94****	0.21 ± 0.11 μV t_17_ = 7.64****	0.12 ± 0.05 μV t_17_ = 10.01****	0.13 ± 0.08 μV t_17_ = 7.08****
syncopated rhythm	0.09 ± 0.06 μV t_17_ = 5.92****	0.08 ± 0.04 μV t_17_ = 9.11****	0.09 ± 0.04 μV t_17_ = 10.08****	0.12 ± 0.03 μV t_17_ = 13.06****
SSEP at non-beat frequencies	0.6 Hz tempo	1.25 Hz tempo	2.5 Hz tempo	3.8 Hz tempo
unsyncopated rhythm	0.03 ± 0.03 μV t_17_ = 4.64****	0.03 ± 0.01 μV t_17_ = 12.56****	0.03 ± 0.01 μV t_17_ = 15.36****	0.03 ± 0.1 μV t_17_ = 12.11****
syncopated rhythm	0.04 ± 0.02 μV t_17_ = 6.13****	0.05 ± 0.02 μV t_17_ = 9.12****	0.04 ± 0.01 μV t_17_ = 14.99****	0.03 ± 0.007 μV t_17_ = 19.50****

On average, the unsyncopated and syncopated rhythms elicited significant SSEP amplitudes at beat and non-beat frequencies (mean ± standard deviation of the amplitude in μV; t-test against zero after baseline subtraction procedure) at the four different tempi. ****p ≤ .0001.

**Table 2:** Values for accuracy (mean signed asynchrony in ms, mean ± standard deviation across participants, the scores closer to zero indicating higher accuracy) and precision (circular variance ranging between 0 and 1, with higher scores indicating higher variance and lower precision) at tapping in synchrony with the perceived beat (task 2).

tapping accuracy	0.6 Hz tempo	1.25 Hz tempo	2.5 Hz tempo	3.8 Hz tempo
unsyncopated rhythm	−264.7 ± 162.9	−156.8 ± 131.3	−20.75 ± 71.17	−14.54 ± 50.97
syncopated rhythm	−100.1 ± 194.6	−1.4 ± 179.8	−34.8 ± 102.6	−20.0 ± 39.7
**tapping precision**	**0.6 Hz tempo**	**1.25 Hz tempo**	**2.5 Hz tempo**	**3.8 Hz tempo**
unsyncopated rhythm	0.22 ± 0.31	0.13 ± 0.23	0.25 ± 0.34	0.60 ± 0.27
syncopated rhythm	0.16 ± 0.24	0.17 ± 0.25	0.44 ± 0.43	0.58 ± 0.32

Note that mean asynchrony and circular variance are inverse measures of accuracy and precision.

## References

[b1] Phillips-Silver J. & Keller P. E. Searching for roots of entrainment and joint action in early musical interactions. Front Hum Neurosci 6, 26 (2012). 22375113 10.3389/fnhum.2012.00026 PMC3288575

[b2] Nozaradan S. Exploring how musical rhythm entrains brain activity with electroencephalogram frequency-tagging. Phil Trans B 369, (1658) 20130393 (2014). 10.1098/rstb.2013.0393 PMC4240960 25385771

[b3] London J. Hearing in time: psychological aspects of musical meter. London. Oxford UP. (2004).

[b4] Jones M. R. Time, our lost dimension: Toward a new theory of perception, attention, and memory. Psychological Review 83, 323–335 (1976) 794904

[b5] Large E. W. Resonating to musical rhythm: Theory and experiment. In Simon Grondin (Ed.) The Psychology of Time. Emerald, West Yorkshire (2008).

[b6] McAuley J.D. In Music Perception, 1st edn (eds. Jones, M.R. ) (Springer Handbook of Auditory Research, 2010).

[b7] Regan D. M. in Human Brain Electrophysiology: Evoked Potentials and Evoked Magnetic Fields in Science and Medicine. (eds. Regan D.M. ) (Elsevier, 1989).

[b8] Nozaradan S., Peretz I., Missal M. & Mouraux A. Tagging the neuronal entrainment to beat and meter. J Neurosci 31, 10234–40 (2011). 21753000 10.1523/JNEUROSCI.0411-11.2011 PMC6623069

[b9] Nozaradan S., Peretz I. & Mouraux A. Selective neuronal entrainment to the beat and meter embedded in a musical rhythm. J Neurosci 32(49), 17572–81 (2012). 23223281 10.1523/JNEUROSCI.3203-12.2012 PMC6621650

[b10] van Noorden L. & Moelants D. Resonance in the perception of musical pulse. J New Music Res 28, 43–66 (1999).

[b11] Parncutt R. A perceptual model of pulse salience and metrical accent in musical rhythms. Music Perception 11, 409–464 (1994).

[b12] Keller P. E., Novembre G. & Hove M. J. Rhythm in joint action: psychological and neurophysiological mechanisms for real-time interpersonal coordination. Phil Trans B 369(1658), 20130394 (2014). 10.1098/rstb.2013.0394 PMC4240961 25385772

[b13] Nozaradan S., Zerouali Y., Peretz I. & Mouraux A. Capturing with EEG the neural entrainment and coupling underlying sensorimotor synchronization to the beat. Cereb Cortex doi: 10.1093/cercor/bht261 (2015). 24108804

[b14] Iversen J. R., Repp B. H. & Patel A. D. Top-down control of rhythm perception modulates early auditory responses. In Dalla Bella S. *et al.* (Eds.) The neurosciences and music III: Disorders and plasticity. Ann NY Acad Sci 1169, 58–73 (2009). 10.1111/j.1749-6632.2009.04579.x 19673755

[b15] Aschersleben G. Temporal control of movements in sensorimotor synchronization. Brain Cogn 48, 66–79 (2002). 11812033 10.1006/brcg.2001.1304

[b16] Repp B. H. Sensorimotor synchronization: A review of the tapping literature. Psychon B Rev 12, 969–992 (2005). 10.3758/bf03206433 16615317

[b17] Berens P. CircStat: A Matlab Toolbox for Circular Statistics. Journal of Statistical Software 31, 10 (2009).

[b18] Pecenka N. & Keller P. E. Auditory pitch imagery and its relationship to musical synchronization. Ann N Y Acad Sci 1169, 282–286 (2009). 19673794 10.1111/j.1749-6632.2009.04785.x

[b19] Mills P. F., van der Steen M. C., Schultz B. G. & Keller P. E. Individual differences in temporal anticipation and adaptation during sensorimotor synchronization. *Timing and Time Perception* In Press (2015).

[b20] Van der Steen M.C., Jacoby N., Fairhurst M. T. & Keller P. E. Sensorimotor synchronization with tempo-chaning auditory sequences: modeling temporal adaptation and anticipation. Brain Res pii. S0006–8993(15), 00087–6 (2015). 10.1016/j.brainres.2015.01.053 25725379

[b21] Galambos R., Makeig S. & Talmachoff P.J. A 40-Hz auditory potential recorded from the human scalp. Proc Natl Acad Sci USA 78, 2643–7 (1981). 6941317 10.1073/pnas.78.4.2643 PMC319406

[b22] Vialatte F. B., Maurice M., Dauwels J. & Cichocki A. Steady-state visually evoked potentials: focus on essential paradigms and future perspectives. Prog Neurobiol 90(4), 418–38 (2010). 19963032 10.1016/j.pneurobio.2009.11.005

[b23] Zhang L., Peng W., Zhang Z. & Hu L. Distinct features of auditory steady-state responses as compared to transient event-related potentials. Plos One 8(7), e69164 (2013). 23874901 10.1371/journal.pone.0069164 PMC3706443

[b24] Capilla A., Pazo-Alvarez P., Darriba A., Campo P. & Gross J. Steady-state visual evoked potentials can be explained by temporal superposition of transient event-related responses. PLoS One 6(1), e14543 (2011). 21267081 10.1371/journal.pone.0014543 PMC3022588

[b25] Chemin B., Mouraux A. & Nozaradan S. Body movement selectively shapes the neural representation of musical rhythm. Psychol Sc 25(12), 2147–59 (2014). 25344346 10.1177/0956797614551161

[b26] Rajendran V., Garcia-Lazaro J., Lesica N. & Schnupp J. Neuronal entrainment to rhythm in the gerbil inferior colliculus. *Proceedings of the “5*^*th*^ *International Conference on Auditory Cortex – Towards a Synthesis of Human and Animal Research”* Magdeburg, Germany (2014).

[b27] Cameron D. J. & Grahn J. A. The influence of culture on rhythm perception, behaviour, and neural entrainment to the beat. Proceedings of the “Rhythm Perception and Production Workshop”. Amsterdam, The Netherlands (2015).

[b28] Gibbings A., Cruse D., Stojanowski B. & Grahn J. A. Attention and presence of a beat modulate neural entrainment to non-repeating rhythms. Proceedings of the “Rhythm Perception and Production Workshop”. Amsterdam, The Netherlands (2015)

[b29] Vuust P. & Witek M. A. Rhythmic complexity and predictive coding: a novel approach to modeling rhythm and meter perception in music. Front Psychol, doi: 10.3389/fpsyg.2014.01111 (2014). PMC4181238 25324813

[b30] Friston K. Functional integration and inference in the brain. Prog Neurobiol 68(2), 113–43 (2002). 12450490 10.1016/s0301-0082(02)00076-x

[b31] Friston K. A theory of cortical responses. Phil Trans B 360(1456), 815–36 (2005). 10.1098/rstb.2005.1622 PMC1569488 15937014

[b32] Rao R. P. & Ballard D. H. Predictive coding in the visual cortex: a functional interpretation of some extra-classical receptive-field effects. Nat Neurosci 2(1), 79–87 (1999). 10195184 10.1038/4580

[b33] Wacongne C. *et al.* Evidence for a hierarchy of predictions and prediction errors in human cortex. Proc Natl Acad Sci USA 108(51), 20754–9 (2011). 22147913 10.1073/pnas.1117807108 PMC3251061

[b34] Friston K. J., Daunizeau J., Kilner J. M. & Kiebel S. J. Action and behavior: a free-energy formulation. Biological Cybernetics 102(3), 227–260 (2010). 20148260 10.1007/s00422-010-0364-z

[b35] Rybarczyk Y. P. & Mestre D. Effect of temporal organization of the visuo-locomotor coupling on the predictive steering. Front. Psychology 3, 239, doi: 10.3389/fpsyg.2012.00239 (2012). PMC3394438 22798955

[b36] Morillon B., Schroeder C.E. & Wyart V. Motor contributions to the temporal precision of auditory attention. Nat Commun 5, 5255 (2014). 25314898 10.1038/ncomms6255 PMC4199392

[b37] Morillon B., Hackett T. A., Kajikawa Y. & Schroeder C. E. Predictive motor control of sensory dynamics in auditory active sensing. Curr Opin Neurosci 31, 230–8 (2015). 10.1016/j.conb.2014.12.005 PMC4898262 25594376

[b38] Merchant H. *et al.* Sensorimotor neural dynamics during isochronous tapping in the medial premotor cortex of the macaque. Eur J Neurosci 41(5), 586–602 (2015). 25728178 10.1111/ejn.12811

[b39] Rossion B. Understanding individual face discrimination by means of fast periodic visual stimulation. Exp Brain Res 232(6), 1599–621 (2014). 24728131 10.1007/s00221-014-3934-9

[b40] Patel A. D. & Iversen J. R. The evolutionary neuroscience of musical beat perception: the Action Simulation for Auditory Prediction (ASAP) hypothesis. Frontiers in Psychol, doi: 10.3389/fnsys.2014.00057 (2014). PMC4026735 24860439

[b41] Moreau P., Jolicoeur P. & Peretz I. Pitch discrimination without awareness in congenital amusia: evidence frome vent-related potentials. Brain Cogn 81(3), 337–44 (2013). 23434917 10.1016/j.bandc.2013.01.004

[b42] Nettl B. An ethnomusicologist contemplates universals in musical sound and musical culture. In Walin *et al.* (Eds.) The origins of music, Cambridge, USA, MIT Press (2000).

[b43] Grahn J. A. & McAuley J. D. Neural bases of individual differences in beat perception. Neuroimage 47(4), 1894–903 (2009). 19376241 10.1016/j.neuroimage.2009.04.039

[b44] Phillips-Silver J., *et al.* Born to dance but beat deaf: a new form of congenital amusia. Neuropsychologia 49(5), 961–9 (2011). 21316375 10.1016/j.neuropsychologia.2011.02.002

[b45] Sowinski J. & Dalla Bella S. Poor synchronization to the beat may result from deficient auditory-motor mapping. Neuropsychologia 51(10), 1952–63 (2013). 23838002 10.1016/j.neuropsychologia.2013.06.027

[b46] Launay J., Grube M. & Stewart L. Dysrhythmia: a specific congenital rhythm perception deficit. Front Psychol, doi: 10.3389/fpsyg.2014.00018 (2014). PMC3913998 24550854

[b47] Penhune V. B. & Doyon J. Cerebellum and M1 interaction during early learning of timed motor sequences. Neuroimage 26(3), 801–12 (2005). 15955490 10.1016/j.neuroimage.2005.02.041

[b48] Ullen F., Forsman L., Blom O., Karabanov A. & Madison G. Intelligence and variability in a simple timing task share neural substrates in the prefrontal white matter. J Neurosci 28(16), 4238–43 (2008). 18417703 10.1523/JNEUROSCI.0825-08.2008 PMC6670305

[b49] Tierney A. & Kraus N. The ability to move to a beat is linked to the consistency of neural responses to sound. J Neurosci 33(38), 1481–8 (2013). 10.1523/JNEUROSCI.0612-13.2013 PMC6618407 24048827

[b50] Pollok B. *et al.* Cortical activations associated with auditorily paced finger tapping. Neuroreport 14, 247–250 (2003). 12598739 10.1097/00001756-200302100-00018

[b51] Chen J. L., Penhune V. B. & Zatorre R. J. Moving on time: brain network for auditory-motor synchronization is modulated by rhythm complexity and musical training. J Cogn Neurosci 20, 226–239 (2008). 18275331 10.1162/jocn.2008.20018

[b52] Merchant H., Pérez O., Zarco W. & Gamez J. Interval tuning in the primate medial premotor cortex as a general timing mechanism. J Neurosci 33(21), 9082–96 (2013). 23699519 10.1523/JNEUROSCI.5513-12.2013 PMC6705035

[b53] Crowe D. A., Zarco W., Bartolo R. & Merchant H. Dynamic representation of the temporal and sequential structure of rhythmic movements in the primate medial premotor cortex. J Neurosci 34(36), 11972–83 (2014). 25186744 10.1523/JNEUROSCI.2177-14.2014 PMC6608467

[b54] Bartolo R., Prado L. & Merchant H. Information processing in the primate basal ganglia during sensory-guided and internally driven rhythmic tapping. J Neurosci 34(11), 3910–23 (2014). 24623769 10.1523/JNEUROSCI.2679-13.2014 PMC6705277

[b55] Nozaradan S. *et al.* Musical entrainment directly recorded in the depth of the human temporal and frontal cortex. Proceedings of the “5^th^ International Conference on Auditory Cortex – Towards a Synthesis of Human and Animal Research”. Magdeburg, Germany (2014).

[b56] Vuust P., Brattico E., Seppänen M., Näatänen R. & Tervaniemi M. Practiced musical style shapes auditory skills. Ann NY Acad Sci 1252, 139–56 (2012). 22524351 10.1111/j.1749-6632.2011.06409.x

[b57] Wallin N. L., Merker B. & Brown S. (Eds.) The origins of music. MIT Press, Cambridge, MA, USA (2000).

[b58] Merchant H. & Honing H. Are non-human primates capable of rhythmic entrainment? Evidence for the gradual audiomotor evolution hypothesis. Front Neurosci, doi: 10.3389/fnins.2013.00274 (2014). PMC3894452 24478618

[b59] Povel D. J. & Essens P. J. Perception of temporal patterns. Music Percept 2, 411–441 (1985). 10.3758/bf03207132 3991313

[b60] Saupe K., Schröger E., Andersen S. K. & Müller M. M. Neural mechanisms of intermodal sustained selective attention with concurrently presented auditory and visual stimuli. Front Hum Neurosci 3, 58 (2009). 20011221 10.3389/neuro.09.058.2009 PMC2791035

[b61] Mouraux A. *et al.* Nociceptive steady-state evoked potentials elicited by rapid periodic thermal stimulation of cutaneous nociceptors. J Neurosci 31, 6079–87 (2011). 21508233 10.1523/JNEUROSCI.3977-10.2011 PMC6632977

[b62] Nozaradan S., Peretz I. & Mouraux A. Steady-state evoked potentials as an index of multisensory temporal binding. NeuroImage 60, 21–8 (2012). 22155324 10.1016/j.neuroimage.2011.11.065

[b63] Fisher N. I. Statistical Analysis of Circular Data. 1^st^ edn (eds Fisher N. I. ) (Cambridge University Press, 1993).

